# A closer look: obsessive-compulsive symptoms among intern nurses amidst COVID-19 pandemic

**DOI:** 10.1186/s12912-024-01872-6

**Published:** 2024-03-28

**Authors:** Mona Metwally El-Sayed, Eman Sameh Abd Elhay, Manal Mohammed Hawash, Hassan Mohammed Sonbol, Samah Mohamed Taha

**Affiliations:** 1 Psychiatric and Mental Health Nursing, Faculty of Nursing, Alexandria University, Alexandria, Egypt; 2 Psychiatric and Mental Health Nursing, Faculty of Nursing, Mansoura University, Mansoura, Egypt; 3 Gerontological Nursing, Faculty of Nursing, Alexandria University, Alexandria, Egypt; 4 Psychiatry, Faculty of Medicine, Mansoura University, Mansoura, Egypt; 5 Psychiatric Nursing and Mental Health, Faculty of Nursing, Mansoura University, Mansoura, Egypt

**Keywords:** Obsessive-compulsive symptoms, COVID-19, Intern nurses

## Abstract

**Background:**

The distinctive circumstances and socio-cultural context in Egypt make it crucial to explore the psychological well-being of intern nurses amid the COVID-19 pandemic, with a specific focus on obsessive-compulsive symptoms. This study aimed to investigate the influence of fear of COVID-19 on obsessive-compulsive symptoms among intern nurses.

**Methods:**

A cross-sectional survey involving 375 randomly recruited intern nurses was conducted. Data collected included the Fear of COVID-19 Scale and the Short Version of the Arabic Obsessive-Compulsive Scale.

**Results:**

A significant relationship was found between the fear of COVID-19 and the severity of obsessive-compulsive symptoms among the participants (*r* = 0.472, *p* = 0.000). A stepwise regression analysis indicated that the fear of COVID-19, living in urban regions, frequency of COVID-19 infection, and increased number of infected family members may contribute to the severity of Obsessive-Compulsive Symptoms with adjusted R2 value = 27.5%.

**Conclusion:**

The findings suggest that the COVID-19 pandemic has had significant psychological impacts on newly qualified nurses during their internship training period, including the manifestation of mental health symptoms such as Obsessive-Compulsive Symptoms. It was observed that urban residents, intern nurses with recurrent COVID-19 infections, and those with more infected family members exhibited a higher severity of OCS. These findings underscore the need for further research to investigate additional factors that may influence OCS severity.

## Introduction

In December 2019, an unknown illness emerged in Wuhan City, China, later identified as COVID-19 caused by the SARS-CoV-2 virus [1]. COVID-19 quickly spread globally, posing a significant threat to human life and health due to its high contagiousness and severity [2]. It has resulted in numerous deaths worldwide, including in Egypt [3]. According to the World Health Organization's report in 2023, Egypt has experienced a significant impact from the ongoing COVID-19 pandemic, with 516,023 confirmed cases and 24,830 deaths recorded in the country [4]. In addition to its deadly effects, COVID-19 has severe psychological consequences, including stress, worry, obsessive thoughts, and depressive disorders [5, 6].

The COVID-19 pandemic has led to the widespread adoption of ritualized washing behaviors and avoidance measures endorsed by organizations like the WHO and implemented by governments [7]. This has unexpectedly caused individuals with modest levels of obsessive-compulsive symptoms (OCS) to exhibit thoughts and behaviors associated with clinical OCD, potentially contributing to a higher prevalence of OCD [8]. Distressing life events, such as the unexpected loss of a family member during the pandemic, have been found to correlate with increased OCS [9]. There is also evidence suggesting an increase in OCS, particularly in individuals with diagnosed OCD, specifically those with Contamination-related OCD or Cleaning-related OCS, during the COVID-19 pandemic [10, 11].

OCD is a chronic mental condition characterized by unwanted thoughts (obsessions) and repetitive behaviors (compulsions). While many individuals engage in repetitive actions in their daily lives, in individuals with OCD, these thoughts and behaviors are more frequent and severe, often significantly impacting their lives [12–14]. OCD ranks as the fourth most prevalent mental disorder globally [15]. The estimated lifetime prevalence of OCD is 2.3%, with a range of 1.1-3.3%. The prevalence of OCD varies based on factors such as age, location, and other factors [12, 16]. Individuals with OCD may exhibit various categories of symptoms, including fixation with contaminated cleaning practices, frequent checking, persistent unwanted or religious thoughts, repetitive hoarding, symmetry, and arranging. These symptoms and behaviors can be erratic, fluctuating over time, or changing their manifestations during the illness [6, 17–19].

The COVID-19 pandemic has had a significant negative impact on global mental health, including among intern nursing students [20]. Studies, particularly in the Arab-speaking countries and MENA region, have shown that a high percentage of medical and nursing students reported experiencing concurrent mental symptoms during the pandemic, with worry and stress being the most common [20, 21]. A study in Saudi Arabia found that OCD symptoms were prevalent in 3.4% of individuals in Asser province [22]. Meanwhile, a study among Iraqi undergraduate medical students during the COVID-19 pandemic found that 43% of the students had probable OCD symptoms [23]. Lastly, in Iran, a study found that 71% of COVID-19-recovered individuals exhibited OCD symptoms [24].

It is a complex condition that can be challenging to diagnose and understand due to symptom overlap with other mental illnesses and diseases. Additionally, individuals may deny or not acknowledge their symptoms and behaviors due to the stigma associated with such mental disorders. This emphasizes the importance of screening for OCD symptoms, particularly in high-risk populations such as intern nursing students [12]. Several researchers have noted that people with obsessive-compulsive symptoms tend to anticipate danger or adverse outcomes more than those without such symptoms, especially during stressful periods. These individuals tend to interpret danger based on the lack of evidence to the contrary rather than the presence of actual danger signals. They are more prone to avoiding risks compared to other groups. One of the most common symptoms of OCS is excessive cleaning, which attempts to control threats by minimizing the risk of harm and ensuring safety [8, 9].

Health crises, such as the COVID-19 pandemic, could potentially exacerbate OC symptoms in younger intern nurses who are already predisposed to such conditions. These external circumstances often act as catalysts for obsessive thoughts and compulsive behaviors. Young interns, who tend to overestimate threats, might harbor numerous concerns or obsessions related to harm, such as the fear of getting infected themselves, inadvertently spreading the virus, or causing harm to others. They might find themselves incessantly having ruminative thoughts and seeking reassurance by obsessively looking up news about COVID-19. Moreover, to alleviate their fear and anxiety, they might fall into the pattern of compulsive handwashing to manage their recurring intrusive obsessive thoughts [17, 19, 22].

Intern nurses in Egypt have played a crucial role in managing the increased number of patients during the COVID-19 pandemic [25]. Before the pandemic, Egyptian hospitals faced challenges such as a high patient load, limited resources, and a staff shortage. The demanding work hours assigned to interns can hurt their mental well-being, leading to increased levels of fear, anxiety, and OCS [26, 27]. The COVID-19 pandemic can further exacerbate these issues, which is particularly significant in Egypt, with its medium-income status and many patients in hospital settings. Thus, this study aimed to investigate the influence of fear of COVID-19 on obsessive-compulsive symptoms among intern nurses.

### Research hypothesis

The researcher hypothesized that intern nurses with a severe level of fear towards COVID-19 would exhibit severe obsessive-compulsive symptoms.

## Methods

### Research design and setting

A cross-sectional survey targeted intern nurses from October 14^th^ to December 20^th^, 2022. The survey encompassed four hospitals affiliated with Alexandria University. These included El-Shatby Hospital for Obstetricians and Gynecologists (A), Main University Hospital (B), El Hadara Orthopedic and Traumatology University Hospital (C), and Smouha University Hospital (D).

### Target participants and sample size calculation

The objective of this study was to scrutinize a cohort of intern nurses who were trained across the four university hospitals. The records indicated 1,002 registered interns (A =498, B =561, C =258, and D = 183). To ascertain an appropriate representative sample size, the EPI INFO 7 software was utilized. The calculation was based on a 5% margin of error, a 95% confidence level, a 50% anticipated proportion, and a population size of 1,002. The software recommended a minimum sample size of 326 participants. However, to account for a potential 10% nonresponse rate, the total sample size was adjusted to 375 participants.

### Inclusion and exclusion criteria

The eligibility criteria of this study included intern nurses who started their internship during the academic year 2022 and exhibited a readiness to participate. Moreover, it was a prerequisite that these participants did not have any diagnosed mental health disorders, were not consuming psychotropic medications, were not involved in ongoing psychotherapy, and had not engaged in the use of substances such as Alcohol, Amphetamines, or psychostimulant drugs.

### Sampling and recruitment process

**Fig. 1:**
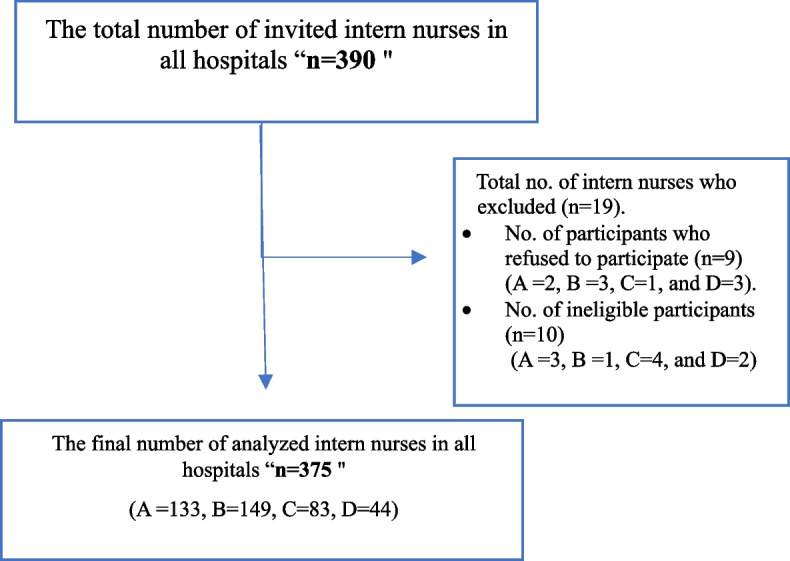
Recruitment flowchart

The study employed a stratified random sampling technique to select participants in proportion to the total number of intern nurses across four hospitals. The research team initially secured approval from the Internship Affairs Committee at the Faculty of Nursing to access the student registration list. This comprehensive list of the participants was then used as the basis for random selection using the Research Randomizer Generation software. Of the 390 invited participants, 9 refused to participate (A =2, B =3, C=1, and D=3), and 10 were ineligible (A =3, B =1, C=4, and D=2). This resulted in a final sample of 375 intern nurses (A =133, B =149, C=83, and D=44) whose responses were analyzed. This final sample was obtained through a rigorous random selection and adherence to the study's eligibility criteria (Fig. 1).

## Study instruments

### A sociodemographic profile for intern nurses

The research team formulated a data collection profile to gather specific information about the intern nurses. This information included their age, gender, place of residence, living arrangements, and frequency of COVID-19 infection. The profile also recorded the number of family members who had previously suffered from a COVID-19 infection. In addition, the data collection profile was designed to capture information on whether the intern nurses were simultaneously working other shifts in private hospitals during their internship training.

### Fear of COVID-19 Scale (FCV-19S)

The FCV-19S is a seven-item instrument that utilizes a 5-point Likert scale, with responses ranging from 1 (strongly disagree) to 5 (strongly agree), to evaluate an individual’s fear of COVID-19 [28]. The total scores can vary from 7 to 35, with higher scores denoting severe fear. The scores are interpreted as follows: 7-15 indicates mild level, 16-25 indicates moderate, and 26-35 indicates severe level. FCV-19S showed a Cronbach's alpha of 0.82, reflecting high internal consistency [28]. The scale was translated into Arabic and then back-translated to ensure its applicability to Arabic-speaking populations. Bilingual Psychiatric Nursing and Mental Health experts reviewed the translated version. A confirmatory factor analysis was conducted to validate the content of the scale post-translation. In our study, the Arabic version of the FCV-19S demonstrated good internal consistency, as evidenced by a Cronbach's alpha of 0.87.

### Short version of Arabic Obsessive-Compulsive Scale (AOCS)

The AOCS is a self-administered tool designed to evaluate symptoms of obsessive-compulsive symptoms [29]. The AOCS includes 25 statements, 20 of which are aimed at identifying OCD symptoms, while the remaining five serve as non-clinical fillers. Participants are asked to rate each item on a 4-point Likert scale, with responses ranging from "No" to "Always." The total score on the AOCS can range from 20 to 80, with a higher score indicating more severe obsessive-compulsive symptoms. The scores are categorized as follows: a score of 20-39 suggests mild OCD symptoms, a score of 40-59 indicates moderate symptoms, and a score of 60-80 signifies severe symptoms. The scale demonstrated a high level of internal consistency, as evidenced by a Cronbach's alpha value of 0.89 [30]. In addition, the AOCS displayed good internal consistency in this study, with a Cronbach's alpha of 0.84.

## Procedure

### Ethical approval

The research was conducted by the Declaration of Helsinki (DoH-Oct2008) guidelines and regulations. Ethical approval was secured from the Research Ethics Committee (REC) at the Faculty of Nursing, El Mansoura University. The Vice Dean of Students' Affairs Faculty of Nursing in Alexandria, Egypt, officially granted permission to conduct the study. The contact details of the intern nurses were obtained from the University's Internship affairs unit. Informed consent was obtained from each participant, who was thoroughly briefed about the study's purpose and assured of their anonymity and the confidentiality of their personal information. Participants were also informed of their right to withdraw from the study at any time without any repercussions, emphasizing that their participation was entirely voluntary.

### Pilot study and tool validity

The researchers developed a sociodemographic profile of intern nurses to gather relevant information. To ensure the content validity of the Fear of COVID-19 Scale (FCV-19S) in Arabic, it was translated into Arabic, then back-translated, and subjected to confirmatory factor analysis. The results of the analysis indicated that the Chi-Square Test of Model Fit was *p*=0.10, the Comparative Fit Index (CFI) was 0.92, the Tucker-Lewis Index (TLI) was 0.93, and the Root Mean Square Error of Approximation (RMSEA) was 0.07. Furthermore, the Arabic Obsessive-Compulsive Scale (AOCS) was adopted and employed in the study. A preliminary study was conducted with 40 intern nurses who were not part of the final sample. This initial study aimed to evaluate the clarity and applicability of the research tools and identify any potential challenges in gathering data. The findings from this pilot study confirmed that the research instruments were accurate, understandable, and suitable for use. Also, the pilot results found that 15% of the participants exhibited a higher OC symptom.

### Data collection

After excluding the participants from the pilot study, a representative sample was selected using a simple random method, following a stratified sampling procedure from the four target hospitals. Interviews were then conducted by a trained psychiatrist using structured questionnaires to gather the necessary data. This was to ensure that the participants did not have any psychiatric disorders, were not taking psychiatric medications or undergoing psychotherapy, and had no history of substance use. Participants were required to provide written informed consent before independently completing the questionnaires. These interviews, each lasting about 10-15 minutes, were conducted individually in a private room in each hospital to ensure privacy. Participants were assured of their anonymity and confidentiality. Participation was entirely voluntary, with no rewards or penalties involved. Intern nurses were informed that they could withdraw without facing any repercussions. To ensure the accuracy and completeness of the data, the researchers meticulously reviewed the responses to the research tool provided by the participants.

### Statistical processing and analysis

The data analysis was conducted using the SPSS program, version 26.0. Initially, the data was coded, cleaned, and checked for normal distribution using the Shapiro-Wilk test. Descriptive statistics were then used to summarize the qualitative data, presenting frequencies and percentages. Descriptive statistics such as mean and standard deviation (SD) were calculated for the quantitative data. The internal consistency of the research instruments, FCV-19S and AOCS, was assessed using Cronbach's alpha. To explore the relationship between the fear of COVID-19 and obsessive-compulsive symptoms, the Pearson correlation coefficient was calculated. Additionally, a linear regression analysis was employed to evaluate the extent to which the fear of COVID-19 explains the variance in obsessive-compulsive symptoms while considering other potential influencing factors.

## Results

Table 1 presents the sociodemographic characteristics of 375 intern nurses. The age groups range from 24 to over 26 years old. Most intern nurses were female (61.07%), resided in urban areas (57.86%), and 83.73% lived with their families. Regarding their history with COVID-19, 14.93% have not been infected, 80.26% have been infected once, 3.20% twice, and 1.61% more than twice. Regarding family members previously Infected with COVID-19, 73.06% had between 1 and 3 family members infected, 22.40% had between 4 and 5, and 4.54% had more than 5—lastly, most intern nurses (90.66%) work in other private hospitals during their internship training.

**Table 1:** Distribution of the participants regarding their sociodemographic data

**Sociodemographic variables**	**Total (*n*=375)**	
	**No**	**%**
**Age**		
24-	140	37.33
25-	128	34.13
26-	107	28.54
**Gender**		
Male	146	38.93
Female	229	61.07
**Residence**		
Urban	217 158	57.86
Rural		42.14
**Living Arrangement**		
Family	314	83.73
Relatives	25	6.66
Alone	36	9.61
**Frequency of COVID-19 infection**		
None	56	14.93
Once	301	80.26
Twice	12	3.20
More than twice	6	1.61
**No. of infected family members**		
1-3	274	73.06
4-5	84	22.40
<5	17	4.54
**Working in other private hospitals during internship training**		
**Yes**	340	90.66
**No**	35	9.34

**Fig. 2:**
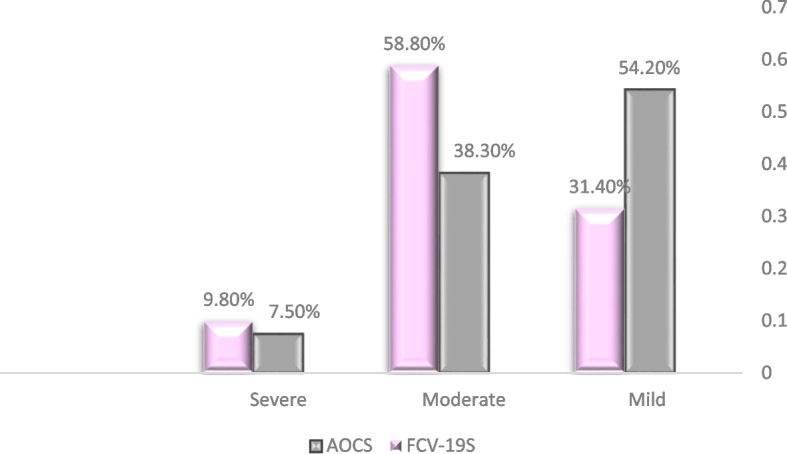
Distribution of the participants according to fear of COVID-19 and OCS severity (*n*=375). AOCS: Short Version of Arabic Obsessive-Compulsive Scale. FCV-19S: Fear of COVID-19 Scale

Figure 2 reveals that most participants (54.2%) reported mild symptoms of OCD. A significant portion (38.3%) reported moderate symptoms. A small percentage (7.5%) reported severe symptoms on the AOCS. The average score on the AOCS scale was 25.81 SD (10.07), suggesting a moderate level of overall symptom severity. The figure also shows that a substantial portion of participants (58.8%) reported moderate fear of COVID-19. A smaller percentage (9.8%) reported severe fear of COVID-19. The average score was 17.96 (5.10), indicating a moderate level of fear related to COVID-19.

Table 2 displays the Pearson correlation coefficient between FCV-19S and AOCS, with *r* = 0.472 and a *p*-value of 0.000. These results indicate a moderate statistically significant positive correlation between the participants' fear of COVID-19 and OCS.

**Table 2:** The correlation coefficient between the fear of COVID-19 and Obsessive-compulsive symptoms severity among the participants (*n*=375)

**Variables**		**FCV-19S**	**AOCS**
**FCV-19S**	Pearson Correlation		
	Sig. (2-tailed)		
**AOCS**	Pearson Correlation	0.472**	
	Sig. (2-tailed)	0.000	

AOCS: Short Version of Arabic Obsessive-Compulsive Scale

FCV-19S: Fear of COVID-19 Scale

r = Pearson correlation

^**^ Correlation is significant at the 0.01 level (2-tailed)

Table 3 reveals that there was no significant difference in OCD symptoms based on age (F=3.578, *p*=0.126), gender (t=0.502, *p*=0.325), or living arrangement (F=1.372, *P*=0.255). However, a significant difference was observed in OCD symptoms based on residence, with urban residents scoring higher on the AOCS scale than rural residents (t=6.335, *p*=0.012). The frequency of COVID-19 infection also showed a significant correlation with OCD symptoms (F=2.588, *P*=0.053), as did the number of infected family members (F=3.472, *P*=0.017). Interestingly, working in other private hospitals during internship training did not significantly affect OCD symptoms (t=4.369, *p*=0.258).

**Table 3:** The relationship between sociodemographic variables and OC symptoms severity among the participants (*n*=375)

Variables	AOCS		Test of significance
	***M***	***SD***	
**Age**			
24-	39.25	9.36	F=3.578 *p*=0.126
25-	38.69	8.36	
26-	38.74	7.15	
**Gender**			
**Female**	37.15	10.35	t= 0.502 *p*=0.325
**Male**	36.31	10.23	
**Residence**			
Urban	40.01	10.34	t= 6.335* *p*=0.012
Rural	31.78	7.026	
**Living arrangement**			
Family	38.83	10.43	F= 1.372 *P*=0.255
Relatives	32.33	5.465	
Alone	36.73	6.871	
**Frequency of COVID-19 infection**			
None	36.00	7.195	F= 2.588* *P*=0.053
Once	39.86	10.59	
Twice	36.73	9.545	
More than twice	41.60	11.85	
**No. of infected family members**			
1-3	37.81	9.269	F= 3.472* *P*=0.017
4-5	40.61	11.44	
<5	49.00	7.071	
Working in other private hospitals during internship training			
**Yes**	42.23	10.69	t=4.369 *p*=0.258
**No**	41.36	9.78	

AOCS: Short Version of Arabic Obsessive-Compulsive Scale

t = Student t test

F= ANOVA test

^*^ Statistically significant at *p* ≤ 0.05

Table 4 presents a stepwise regression model examining the impact of fear of COVID-19 and other covariates on OCD severity. In the first step, 'FCV-19S' explained 16.5% of the variance in OCD severity, with a significant impact (*p* < 0.001). In the second step, 'Residence' is added to the model, increasing the explained variance to 18.9%, with both variables showing a significant impact. The third step introduces 'Frequency of COVID-19 infection', increasing the explained variance to 23.2%. Finally, in the fourth step, 'No. of infected family members’ was added, resulting in a total variance of 27.5% explained by the model. All variables in the final model have a significant impact (*p* < 0.05).

**Table 4:** A hierarchical regression analysis between OCD Symptoms Severity, fear of COVID-19, and other covariates (*n*=375)

	AOCS					
	B	Beta	t	p	95% CI	
					**LL**	**UL**
**Step1**						
**FCV-19S**	17.874	0.515	24.185	<0.001^*^	15.351	18.861
**R**^**2**^**= 0.165, Adjusted R**^**2**^= 0.164, F = 584.921, *p* <0.001^*^						
**Step2**						
**FCV-19S**	17.874	0.517	24.341	<0.001^*^	15.351	18.861
**Residence**	5.552	0.064	2.999^*^	0.003^*^	9.184	1.921
**R**^**2**^**= 0.189, Adjusted R**^**2**^= 0.188, F = 298.396, *p* <0.001^*^						
**Step3**						
**FCV-19S**	17.874	0.517	24.398	<0.001^*^	15.351	18.861
**Residence**	5.787	0.066	3.129	0.002^*^	9.415	2.159
**Frequency of COVID-19 infection**	2.280	0.059	2.771	0.006^*^	0.666	3.894
**R**^**2**^**= 0.232, Adjusted R**^**2**^= 0.231, F = 202.308, *p* <0.001^*^						
**Step4**						
**FCV-19S**	16.952	0.511	23.935	<0.001^*^	15.351	18.861
**Residence**	5.492	0.063	2.968	0.003^*^	9.122	1.862
**Frequency of COVID-19 infection**	2.243	0.058	2.730	0.006^*^	0.631	3.855
**No. of infected family members**	2.832	0.053	2.481	0.013^*^	0.593	5.071
**R**^**2**^**= 0.275, Adjusted R**^**2**^= 0.273, F = 153.752, *p* <0.001^*^						

AOCS: Short Version of Arabic Obsessive-Compulsive Scale

F (ANOVA)

R2: Coefficient of determination

B: Unstandardized Coefficients

Beta: Standardized Coefficients

t: t-test

*LL* Lower limit, *UL* Upper Limit, FCV-19S: Fear of COVID-19 Scale

^*^: Statistically significant value at *p* ≤ 0.01

^a^Dependent variable (Obsessive Compulsive Symptoms Severity)

^b^Independent variable (Fear of COVID-19, residency, Frequency of COVID-19 infection, and No. of infected family members)

## Discussion

The COVID-19 pandemic has made hospital clinical placements challenging and frightening for intern nurses, who fear incubating the virus and spreading it to their loved ones and colleagues [31]. Intern nurses have been deployed to COVID-19 departments, taking on roles typically filled by registered nurses. Traumatic experiences or stressful life events are risk factors for obsessive-compulsive and related disorders [32]. The COVID-19 pandemic, with its serious health risks and unpredictability, may exacerbate pre-existing symptoms and trigger new ones in those predisposed to OCD [10, 33]. This study aimed to investigate the influence of COVID-19 fear on obsessive-compulsive symptoms among intern nurses.

The study found that many participants exhibited a modest COVID-19 fear. This could be attributed to these intern nurses' often being asked to work in high-risk areas such as isolation units, emergency departments, and intensive care units where COVID-19 patients were treated. This could increase anxiety among intern nursing students due to misconceptions or a lack of understanding about diseases and infection control procedures relevant to their clinical practice. As a result, students may become more fearful, and diseases may spread more rapidly. The lack of safety precautions in hospitals and the intern nurses' insufficient experience in a COVID-19 environment could further exacerbate these fears [34, 35].

Several studies have highlighted the challenges faced by nursing interns during the COVID-19 pandemic. Key issues include a lack of knowledge and skills, concerns about family, disorientation, and stress from various factors such as fear of making mistakes, handling emergencies, and dealing with death [36, 37]. Mostafa and Abu Zead (2021) found that most nursing interns initially showed reluctance to work in isolation units and care for COVID-19 patients due to fear of infection [38]. Aslan and Pekince (2021) [39] and Rana et al. (2022) [31] found that 61.1% and 68.1% of students had a moderate fear of contracting the infection, respectively.

The current study indicates that most participants displayed mild to moderate levels of OCD, with an average score of 25.81 on the AOCS scale, suggesting a moderate level of overall symptom severity. This could be related to those intern nurses beginning their clinical practice amidst the COVID-19 pandemic. They were faced with a multitude of challenges, such as executing complex and demanding nursing procedures, operating in high-stress and infection-prone settings, managing patients with diverse diagnoses, including those in terminal stages, and dealing with anxious family members. These stressors could adversely affect their cognitive patterns, triggering stress-related schemas in these young students and potentially leading to an escalation in obsessive-compulsive symptoms. This aligns with other studies conducted in Egypt. For instance, a study conducted in Cairo found an incidence of OCD of 2.5% among 1,000 undergraduates attending a university clinic [40]. Another study revealed that the prevalence of Obsessive-Compulsive Symptoms (OCS) is 38.7% in secondary schools [41]. In a study conducted by Taher et al. (2021) in Iraq, it was reported that a significant number, 1153 (70.1%), reported experiencing mental symptoms, with worry and stress being the most common, affecting 674 (25.9%) and 617 (23.7%) of the students respectively. Interestingly, 707 (43%) of the students exhibited potential OCD symptoms that warrant further investigation. The most common symptom was unpleasant thoughts, experienced by 51.8% of the students. Surprisingly, the scores for washing and contamination were relatively low at 14% and 19.4%, respectively, while the least common symptom was the repetition of specific numbers, reported by only 8% of the students [23].

A study by Wang et al. (2023) involving 496 nurses in China found that the mean score of obsessive-compulsive symptoms was 47.21, with 94% of participants scoring above the median of 36, indicating a higher prevalence of OCD symptoms [42]. Ergenc et al. (2020) found that healthcare workers in the COVID-19 section had significantly higher rates of anxiety, depression, and obsessive-compulsive disorders compared to the control group [43]. The pandemic has led to an increase in OCD washing symptoms [44] and a greater incidence of OCD symptoms among healthcare workers compared to the public [45]. A longitudinal study by Pan et al. (2021) found that the pandemic did not appear to worsen the high levels of symptoms in those with severe or persistent mental health disorders. However, those with no or less severe mental health disorders experienced higher levels of depressive symptoms, anxiety, worry, and loneliness [46]. A rapid scoping review by Grant et al. (2022) found that a significant proportion of people with OCD experienced symptoms of worsening during the pandemic, particularly during initial restrictions. They also raised important questions about how exposure-based therapy should be modified during pandemics, how to reduce the risk of OCD aggravation in vulnerable people because of public health messaging, and whether COVID-19 infection is associated with OCD symptoms [11].

The present study has identified a significant connection between the fear of getting infected with COVID-19 and the occurrence of obsessive-compulsive symptoms among nursing interns. The adjusted R-squared value of 0.165 indicates that fear can explain 16.5% of the variance in OCS. This outcome can be understood in the context of Hobfoll (2001) theory, which posits that prolonged stress and fear can lead to a depletion of resources [47]. The fear associated with the pandemic acts as a significant barrier to effective coping mechanisms, potentially exacerbating OC symptoms. The fear and anxiety triggered by the COVID-19 crisis can amplify existing fears of contamination in some interns, leading to an increase in OC symptoms and compulsive behaviors. For these interns, the coronavirus can become a pervasive thought, with fears of touching contaminated surfaces or coming too close to others. The fear of COVID-19 may also make them more compliant with pandemic restrictions, such as social isolation, which could further negatively impact their OC symptoms.

However, these findings were inconsistent with a study by Ji et al. (2020) that examined the impact of fear of adverse events on Yale-Brown Obsessive-Compulsive Scale (Y-BOCS) scores in a COVID-19 fear-inducing environment. They found that the prevalence of possible OCD was significantly higher during the early stages of the pandemic. Still, there was a significant decrease in Y-BOCS score, anxiety level, quarantine level, and intensity of fear of COVID-19 during the middle and late stages of the pandemic. Participants with probable OCD reported higher levels of fear than those with a lower Y-BOCS score (<16), indicating that an interaction between the environment (COVID-19 pandemic) and psychology (fear and anxiety) may be involved in OCD. This suggests that fear of adverse events may play a role in the etiology of OCD [48]. A rapid scoping review by Grant et al. (2022) found that a significant proportion of people with OCD experienced symptoms worsening during the pandemic, particularly during initial restrictions. They also raised important questions about how exposure-based therapy should be modified during pandemics, how to reduce the risk of OCD aggravation in vulnerable people because of public health messaging, and whether COVID-19 infection is associated with OCD symptoms [11].

The study found that intern nurses residing in urban areas demonstrated a higher prevalence of Obsessive-Compulsive (OC) symptoms compared to those living in rural areas. However, this finding is contradicted by Cao et al. (2020), who suggested that urban living could cause worries and stress among university students [49]. The increased fear of COVID-19 in urban areas is thought to be due to greater exposure to media, supported by Garfin et al. (2020), who suggested that repeated exposure to media content related to the outbreak can result in heightened stress responses, thereby affecting both physical and mental health [50]. On the other hand, the robust social networks, community cohesion, and strong family support systems prevalent in rural areas may protect against worries throughout demanding times by providing emotional support, practical help, and reassurance to intern nurses experiencing COVID-19 fear and obsessive-compulsive symptoms. Family members can help interns cope with challenges, reduce stress, and alleviate anxiety. Additionally, family members can play a crucial role in helping interns make informed decisions about their work and take appropriate precautions to protect themselves from COVID-19. Ultimately, family support can help nursing interns feel more confident and secure in their role, positively impacting their overall performance and patient care. This is supported by earlier studies, which suggested that residing with parents and having the family's backing can shield against the fear of external stressors [51, 52].

The research findings indicate a notable link between repeated COVID-19 infections and the intensity of OCS. This suggests that individuals who have experienced multiple bouts of COVID-19 may be more susceptible to severe OCD symptoms. This could be due to the repeated stress and anxiety associated with recurrent infections, which may exacerbate OCD symptoms. Similarly, an increased number of infected family members was also found to influence OCD symptoms significantly. This could be attributed to the added emotional stress and worry about the health and well-being of loved ones, which could exacerbate OCD symptoms. The subjects in the study conducted by Dehghani et al. (2023) had an average score on the obsessive-compulsive disorder scale of 32.90, SD= 19.87, while the average score for fear of coronavirus was 16.82, SD=5.79. Among the dimensions of OCD, contamination had the highest score of 9.04, SD=5.46, while stealing had the lowest score of 0.10, SD=0.49. Individuals with a history of infection had a significantly higher mean fear of COVID-19 than those without (*P* = 0.002). As the fear of coronavirus scale score increased, the score of obsessive-compulsive disorders also increased, except for the stealing dimension (*P* < 0.001) [53]. These studies highlight the significant impact of the COVID-19 pandemic on mental health, particularly among intern nurses. They underscore the need for targeted interventions and support systems to help these individuals cope with the ongoing crisis.

## Limitations of the study

While the study provides valuable awareness of the relationship between the fear of COVID-19 and the severity of obsessive-compulsive symptoms among intern nurses, there are several potential limitations to consider. The reliance on self-reported data could introduce bias, as participants may not accurately recall or may underreport or overreport their symptoms. As a cross-sectional study, it provides a snapshot at a single point in time and does not allow for examining changes over time or determining causality. There may also be unmeasured confounding variables that could influence the observed relationship, such as personal resilience, pre-existing mental health conditions such as anxiety or depression, history of traumatic events, social support systems, coping mechanisms, and personality traits. The study's cultural context may limit the findings' generalizability to other cultural settings.

## Conclusion and recommendations

The study's findings suggest that the COVID-19 pandemic has had significant psychological impacts on new nurses during their internship training period, including the manifestation of mental health symptoms such as obsessive-compulsive symptoms. It was observed that urban residents, intern nurses with recurrent COVID-19 infections, and those with more infected family members exhibited a higher severity of OCS. A notable correlation was found between the fear of COVID-19 and the severity of OCS among the intern nurses, indicating that fear of the virus may exacerbate OCS. However, it is essential to note that there are likely other contributing factors that need to be accounted for in this model that may also influence the severity of OCS. These findings underscore the need for further research to investigate additional factors that may influence OCS severity.

### Nursing implications

The research underscores the importance of providing new interns with mental health support in internship training units. This could include counseling, stress management workshops, peer support groups, and telepsychiatry consultations. Training programs that arm nurses with effective coping strategies could enhance mental health outcomes and overall well-being. This is particularly crucial for those residing in urban areas, individuals infected multiple times, and their family members.

## Data Availability

No datasets were generated or analysed during the current study.
